# Graphene Quantum Dots Improved “Caterpillar”-like TiO_2_ for Highly Efficient Photocatalytic Hydrogen Production

**DOI:** 10.3390/ma14185354

**Published:** 2021-09-16

**Authors:** Jing Ma, Lihua Chu, Yanjiao Guo, Changxu Sun, Hao Yan, Ze Li, Meicheng Li

**Affiliations:** State Key Laboratory of Alternate Electrical Power System with Renewable Energy Sources, North China Electric Power University, Beijing 102206, China; JingMa@ncepu.edu.cn (J.M.); ncepugyj@126.com (Y.G.); cxsun@ncepu.edu.cn (C.S.); hyan@ncepu.edu.cn (H.Y.); zli@ncepu.edu.cn (Z.L.); mcli@ncepu.edu.cn (M.L.)

**Keywords:** GQDs, TiO_2_ caterpillar, photocatalysts, hydrogen production, water-splitting

## Abstract

Photocatalytic water splitting for hydrogen production via heterojunction provides a convenient approach to solve the world crises of energy supply. Herein, graphene quantum dots modified TiO_2_ hybrids (TiO_2_-GQDs) with a “caterpillar”-like structure exhibit stronger light absorption in the visible region and an enhanced hydrogen production capacity of about 3.5-fold compared to the pristine TiO_2_ caterpillar. These results inferred that the addition of GQDs drastically promotes the interfacial electron transfer from GQDs to TiO_2_ through C–O–Ti bonds via the bonding between oxygen vacancy sites in TiO_2_ and in-plane oxygen functional groups in GQDs. Using a “caterpillar”-like structure are expected to provide a new platform for the development of highly efficient solar-driven water splitting systems based on nanocomposite photocatalyst.

## 1. Introduction

Global energy inequality, climate change, and consumption of fossil fuels have prompted researchers to turn their attention toward renewable energy [1]. Hydrogen energy is a new green pollution-free energy with many advantages, including high calorific value, easy storage and transportation, etc. [2,3]. Since Fujishima and Honda reported the photoelectrochemical hydrogen evolution process from water in 1972, efficient photoconversion of water to hydrogen has been a long-term goal in the field of photocatalysis [4,5]. Among all the photocatalysts, titanium dioxide (TiO_2_) is considered one of the most promising photocatalysts due to its relatively low price, environmental friendliness, superior photocatalytic performance, and long-term stability [6,7]. Wang et al. [8] summarized photocatalytic properties of anatase/bronze TiO_2_ in 2020. However, due to the wide band gap energy of TiO_2_ (anatase 3.2 eV), its light absorption range is mainly limited to the ultraviolet (UV) region, and the separation efficiency of photogenerated electron-hole pairs is low, which hinders the development of TiO_2_ in the field of photocatalysis [9]. In order to improve the photocatalytic performance, heterogeneous catalysts that combine semiconductor quantum dots (QDs) with TiO_2_ have been proposed, such as CdS, CdSe, PbS, etc. [10,11].

Among many semiconductor QDs, graphene quantum dots (GQDs) are monolayer or multilayer graphene nanosheets with a transverse size of less than 100 nm [12]; due to its distinctive electronic and optical properties, it has become a research hotspot as photocatalytic nanomaterials [13]. Because of the existence of quantum confinement effect, zero dimensional GQDs have discrete electron energy levels, which is different from nanosheets and nanoribbons [14,15]. In particular, the energy gap of GQDs is adjustable, and it can absorb sunlight of any wavelength by changing its size [16]. Increased studies are trying to apply GQDs to the field of photocatalysis, for example, in 2020, Chen et al. [17] discussed GQDs modified g-C_3_N_4_ as a photocatalyst and explored its modification mechanism, and Wei et al. [18] constructed an efficient photocatalyst based on Zn-MOF@GQDs heterostructures. Moreover, it has been proposed that coupling GQDs with TiO_2_ has been attempted to improve the charge separation and visible light absorption of the TiO_2_ [19,20,21], but the specific charge transfer mechanism has not been mentioned in most literature.

Herein, TiO_2_-GQDs “caterpillar”-like nanoarchitecture hybrids were synthesized and C–O–Ti bond is formed via the connection between the oxygen vacancy in TiO_2_ and in-plane oxygen functional groups in GQDs during the ultrasound, thus producing the enhancement of photocatalytic performance in heterostructures. We found that introduction of GQDs on TiO_2_ caterpillar was essential to boosting light-driven water splitting for H_2_ production, and the working mechanism was elucidated by the schematic diagram of interfacial charge transfer. In work, the inherent defects of TiO_2_, such as only absorbing UV light and fast recombination of photogenerated carriers, were improved by compounding with GQDs. These results provide clues for the construction of hybrid nanocatalysts with special morphology of QDs and metal oxides semiconductor, which can be applied in optics, catalysis, and the environment.

## 2. Materials and Methods

### 2.1. Chemicals

Concentrated sulfuric acid (98% H_2_SO_4_) and concentrated nitric acid (68% HNO_3_) were obtained from Sinopharm (Shanghai). Sodium hydroxide (NaOH, 96%) was purchased from Aladdin Bio-Chem Technology Co. (Shanghai, China). Titanium dioxide (TiO_2_, 99%) for caterpillar, and sodium sulfate (Na_2_SO_4_) were procured from InnoChem Technology Co., Ltd. (Beijing, China). Acetic acid (CH_3_COOH, 99%), AgNO_3_, tetrabutyl titanate (TBOT), and H_2_PtCl_6_ were purchased from Sigma-Aldrich Trading Co., Ltd. (Shanghai, China). Nafion solution (perfluorosulfonic acid) was gained from Dupont China Holding Co., LTd. (Shenzhen, China).

### 2.2. Fabrication of TiO_2_-GQDs Caterpillar Hybrids

**Figure 1:**
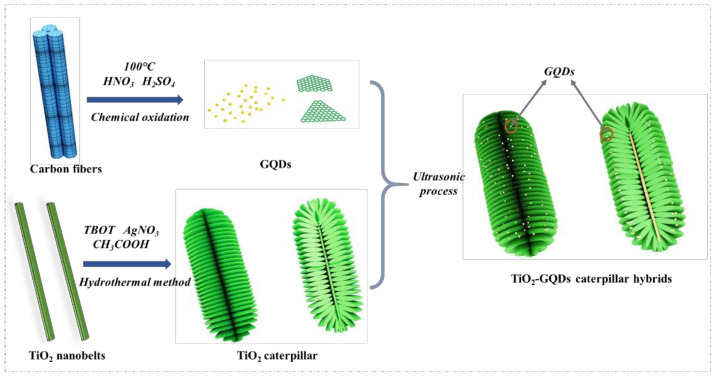
Synthesis of the TiO_2_-GQDs caterpillar hybrids nanoarchitecture.

The GQDs were prepared by chemical oxidation, as reported by Peng et al. [22]. Micrometer-sized pitch-based carbon fibers (0.3 g) were added into a mixture of concentrated H_2_SO_4_ (60 mL) and HNO_3_ (20 mL) with stirring in an oil bath at a temperature of 100 °C for 24 h. After cooling, the mixture was diluted with deionized (DI) water (600 mL), and the pH was adjusted to 8 with NaOH. Finally, the GQDs solution was further dialyzed in a dialysis bag (retained molecular weight: 2000 Da) for 3 days. The TiO_2_ caterpillar nanoarchitecture was prepared by the hydrothermal method [23]. TiO_2_ nanobelts were first prepared and then placed into the mixed solution with 0.5 mL TBOT, 0.1 g AgNO_3_, and 40 mL CH_3_COOH. After stirring, the solution was transferred to a high pressure reactor which then was heated in a drying oven at 150 °C for 8 h. Subsequently, the product was calcined in a muffle furnace at 450 °C for 1 h to obtain the TiO_2_ caterpillar. In order to obtain the TiO_2_-GQDs caterpillar hybrids, appropriate amount of TiO_2_ caterpillar and GQDs are mixed with ultrasound and stirring for 30 min, finally dried into a powder. All the above steps are conducted in an air atmosphere. The preparation process is shown in Figure 1.

### 2.3. Characterization and Electrochemical Measurements

The morphology of samples was characterized by scanning electron microscopy (SEM) (SIRION 200, FEI, Hillsboro, OR, USA) and transmission electron microscopy (TEM) using a Tecnai G2 F20 (FEI, Hillsboro, OR, USA). Atomic force microscopy (AFM) (Autoprobe CP-research, Veeco, Plainview, NY, USA) was performed to observe the surface roughness. X-ray diffraction (XRD) analysis (D8 focus, Bruker, Germany) and Raman spectra (Raman-11, Nanophoton, Osaka, Japan) were used to study chemical compositions and structures. X-ray photoelectron spectroscopy (XPS) and valence band XPS (VB XPS) were conducted on ESCALAB 250Xi (Thermo Fisher Scientific, Waltham, MA, USA). Optical absorption spectra were studied by UV-visible diffuse reflectance spectroscopy (DRS) from 200 to 800 nm wavelength (UV-2600, Shimadzu, Kyoto, Japan). Photoluminescence (PL) spectroscopy (FLS980) was used to study the luminescence characteristics of GQDs. Mott–Schottky (M–S) curve measurements were performed on a CHI660E workstation (CH Instruments, Shanghai, China) based on a conventional three-electrode framework with a Pt plate as counter electrode, saturated calomel electrode (SCE) as the reference electrode, and 0.05 M Na_2_SO_4_ aqueous solution as the electrolyte. Specifically, the working electrodes were prepared by the slurry made of photocatalyst, absolute ethanol, and Nafion solution onto the pretreated fluorine-doped tin oxide (FTO) glass by the dip-coating method, and then dried to form a film electrode.

### 2.4. Photocatalytic Experiments

The hydrogen production rate under illumination measured the photocatalytic activities of samples. A certain amount of photocatalysts with 5 wt% of Pt (H_2_PtCl_6_ as a precursor) were dispersed in a top-irradiated photoreactor mixed with DI and methyl alcohol as a sacrificial agent. The reactor was sealed and slowly loaded into a photocatalytic hydrogen production system. Subsequently, the reaction solution was thoroughly degassed before photocatalytic measurement. To adjust the cooling water flow rate, the water temperature is generally set at 5.0 °C. Nitrogen gas was supplied, and a 300 W Xenon light source (66485-300XF-R1, Newport, RI, USA) was turned on. After the experiment started, the system collected and tested H_2_ every half hour for about 6 h. The peak area of H_2_ obtained from the test results was substituted into the standard curve equation, and the volume of H_2_ was calculated. Finally, the hydrogen production amount was calculated by conversion.

## 3. Results and Discussion

### 3.1. Morphology Study

**Figure 2:**
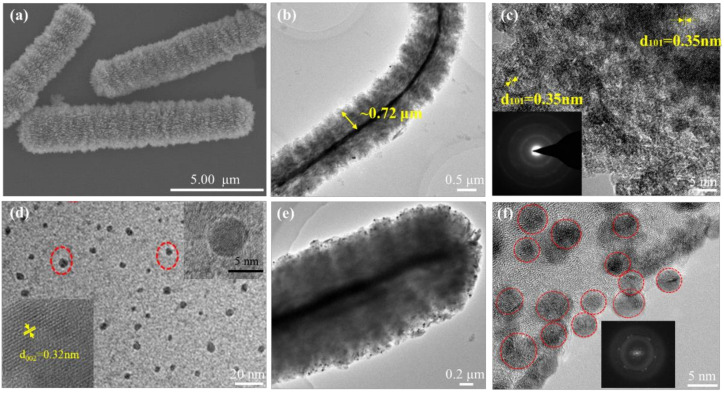
(**a**) SEM, (**b**) TEM and (**c**) HRTEM images of pristine TiO_2_. (**d**) TEM image of GQDs showing an average size of ∼5 nm. The inset shows the HRTEM lattice image. (**e**) TEM and (**f**) HRTEM images of TiO_2_-GQDs caterpillar hybrid. The inset in image in (**f**) is the FFT pattern of GQDs.

The size and morphologies of the pristine TiO_2_ caterpillar, GQDs and their hybrids were characterized by SEM, TEM and high resolution TEM (HRTEM). The SEM image of the pristine TiO_2_ in Figure 2a reveals a “caterpillar”-like morphology, the 3D bunchy architecture consisted of considerable ultrathin nanosheets. The detailed framework of the TiO_2_ caterpillar was further recorded by TEM image (Figure 2b). It is observed that the nanosheets are as thin as the nanometer level, while the length of caterpillar nanoarchitecture is ~10 μm, and the radius is ~0.72 μm. Figure 2c shows the HRTEM image of the pristine TiO_2_ and its corresponding selected area electron diffraction (SAED) pattern. Interplanar spacing of around 3.5 Å assigned to the (101) plane of TiO_2_ crystal [24] and the SAED pattern shows its polycrystalline properties. GQDs with average size ∼5 nm in diameter and a lattice spacing of 0.32 nm corresponding to the (002) plane are displayed in Figure 2d. As Figure 2e reveals, the GQDs attach to the surface and inside of the TiO_2_ nanosheets in the caterpillar nanoarchitecture, which was not destroyed after the ultrasonic treatment. To further confirm the coupling of the GQDs with TiO_2_, Figure 2f indicates the HRTEM lattice image of the TiO_2_-GQDs hybrid sample, where the lattices of both TiO_2_ and GQDs are well-defined, revealing the strong attachment of the GQDs over the TiO_2_ surface. The average diameter of GQDs shown with dotted circles is about ~5 nm, which is consistent with the result of Figure 2d. The inset in Figure 2f shows the corresponding fast Fourier transform (FFT) pattern of GQDs confirming the sp^2^ hybridized, hexagonal graphitic structure [25]. As mentioned above, “caterpillar”-like morphology refers to substantial nanosheets attached to the nanobelt existing in the center of 3D bunchy architecture, which possesses a large surface area and may provide more exposed active sites and decrease the transmission distance of photo-excited electron-hole pairs.

### 3.2. Characterization of GQDs

**Figure 3:**
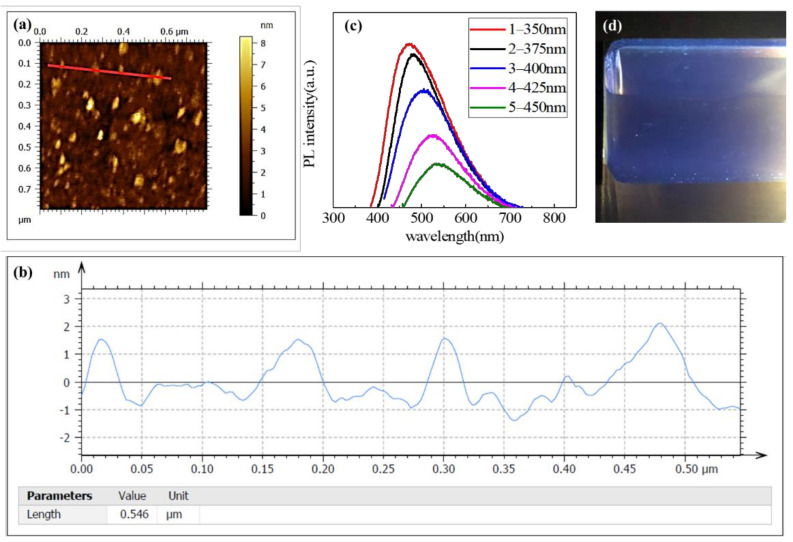
(**a**) AFM image and (**b**) line profile measurement for GQDs. (**c**) PL spectrum of GQDs with different excitation wavelengths, (**d**) luminescence characteristics of the GQDs solution under UV light (365 nm).

To further study the properties of the GQDs, AFM analysis has been conducted. The AFM image of GQDs and the particle size distribution are shown in Figure 3a,b. The bright spots indicate the existence of nanometer GQDs. Line profile measurement for GQDs is obtained by Gwyddion software tool [26], indicating that the GQDs sample has an average height of 0.8–1.2 nm, which corresponds to 2–3 graphene layers. Furthermore, the optical properties of GQDs were studied by the PL technology with different excitation wavelengths. As shown in Figure 3c, with the excitation wavelength increasing from 350 to 450 nm, the PL peaks red-shift from 470 to 530 nm and cause a decrease in the PL peak intensity elucidating the excitation-dependent PL behavior of GQDs, which reveals a Stokes shift and corresponds to the confinement effect [27]. The full-width at half-maximum (FWHM) of PL peaks is about 100 nm, which is a typical value of carbon-based quantum dots [26]. Figure 3d shows that GQDs solution emits bright blue fluorescence under 365 nm UV light, which is caused by the down conversion characteristics [28]. These PL results confirm that the quantum dots have completely localized electronic states and unique optical properties, which will give full play to their advantages when coupled with TiO_2_.

### 3.3. XRD and Raman Spectroscopy Studies

**Figure 4:**
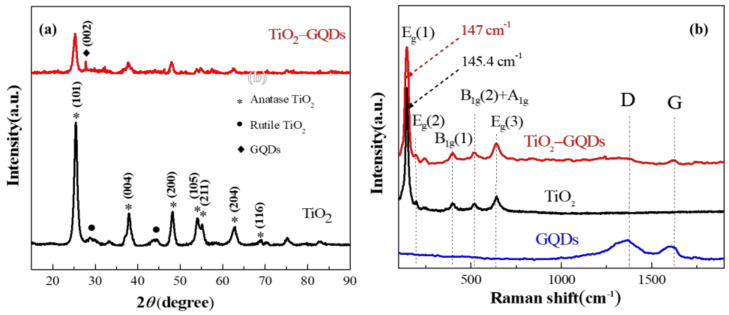
(**a**) XRD results of pristine TiO_2_ and TiO_2_-GQDs caterpillar hybrid. The symbols of “*”, “•”, and “♦” correspond to the anatase TiO_2_, rutile TiO_2_, and GQDs, respectively. (**b**) Characteristic Raman spectra of pristine TiO_2_ caterpillar, GQDs and TiO_2_-GQDs caterpillar hybrid. The Raman of TiO_2_ and GQDs are marked with standard symbols. To ensure clarity, the curve moves vertically.

The prepared pristine TiO_2_ caterpillar and hybrids of GQDs and TiO_2_ show excellent crystallinity, as evidenced by XRD results (Figure 4a). In the pristine TiO_2_, the intense peaks at 25.31°, 37.79°, and 48.04° correspond to the anatase phase (101), (004), and (200) planes of TiO_2_ (JCPDS Card no. 21-1272), respectively. In addition, it can be seen that there is a characteristic peak of rutile TiO_2_ (JCPDS Card no. 21-1276), but the peak intensity is weak. In the TiO_2_-GQDs caterpillar hybrid sample, the obvious peak at 26.3° corresponds to the (002) plane of sp^2^ carbon in GQDs. No other diffraction peaks related to GQDs were observed in the XRD pattern due to the low concentration of GQDs in the hybrid. As compared to pristine TiO_2_ caterpillar, the decrease in crystallinity of hybrid may be due to the ultrasonic treatment that causes defects or disorder. Raman spectroscopy can be used to characterize semiconductor nanostructures and graphite based materials. Using Raman spectroscopy, the changes in the crystallinity/disorder, defects, and internal stress of the samples can be observed. The Raman spectra collected from the GQDs, TiO_2_ caterpillar, and TiO_2_-GQDs caterpillar hybrids in the region of 100–1900 cm^−1^ are shown in Figure 4b. Both the TiO_2_ and TiO_2_-GQDs caterpillar hybrid samples show the characteristic Raman bands of anatase TiO_2_, such as three *E_g_*, two B_1g_, and one A_1g_ modes [29], indicating that ultrasonic treatment will not change the phase of TiO_2_. The strongest *E_g_*(1) mode that corresponds to the symmetric lattice angular vibration of the O–Ti–O bond is the characteristic peak of anatase TiO_2_, which is consistent with the literature [30]. It can be seen that the Raman *E_g_*(1) centers of TiO_2_ and TiO_2_-GQDs hybrid are 145.4 cm^−1^ and 147 cm^−1^, respectively, and the peak shifts by 1.6 cm^−1^, indicating the strong interaction between the GQDs and TiO_2_ in TiO_2_-GQDs hybrid. In the case of TiO_2_-GQDs caterpillar hybrid, the broadening in Raman line shape is possible due to the lattice strain caused by covalent bond, such as C–O–Ti. The D and G peaks observed on Raman spectra are typical to graphene-based materials. The first Raman peak in GQDs, the G band is an in-plane vibration of C=C sp^2^ hybridized carbon atoms, which belongs to the E_2g_ irreducible representation. In addition to the identified G peak, the defect-induced D peak which originates from the “unorganized” carbon associated with the defects or edges of graphene [24,31]. The type of edge configuration of GQDs can be determined by the intensity of D band. The D band is prominent for the armchair edges but absent for the zig-zag edges of GQDs. As a result, the D band of TiO_2_-GQDs is inconspicuous, such that the GQDs edges are zig-zag in the hybrids. In order to estimate the relative contributions of the in-plane and the edge states, the Raman intensity ratios of the D band to G band (ID/IG) [32] in GQDs and TiO_2_-GQDs hybrid were calculated with the ratios of 1.28 and 1.15, respectively. The results show that the ID/IG ratios of the samples change only slightly, indicating that the GQDs have a uniform edge configuration. It shows that despite the TiO_2_-GQDs hybrid being formed, the GQDs edge type did not change, which implies that the change mainly occurs in the in-plane oxygenated functional (epoxy) groups of GQDs. The epoxy groups in the edge and in-plane are redistributed and provide the in-plane epoxy C–O in the process of ultrasonic treatment, which is directly related to the bonding of TiO_2_ caterpillar with the GQDs through the connection of basal plane C–O to the Ti in TiO_2_.

### 3.4. XPS Studies

**Figure 5:**
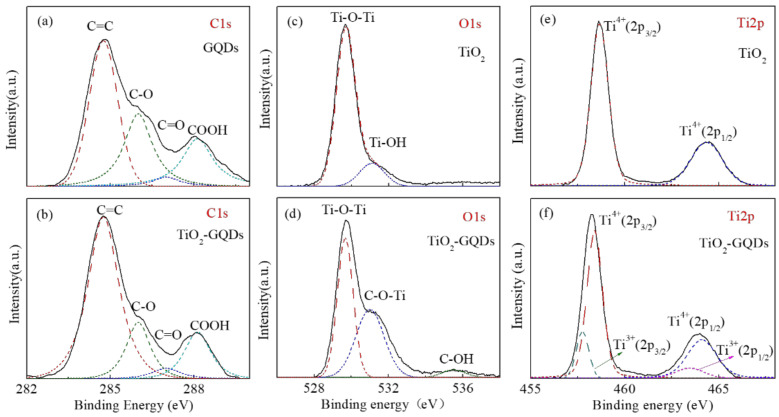
High-resolution XPS spectra of samples: (**a**,**b**) C1s spectra for GQDs and TiO_2_-GQDs caterpillar hybrid, (**c**,**d**) O1s, and (**e**,**f**) Ti2p for TiO_2_ caterpillar and TiO_2_-GQDs caterpillar hybrid.

In order to further study the covalent bond in the hybrid, XPS was performed on the TiO_2_ caterpillar, GQDs, and TiO_2_-GQDs hybrids. Figure 5a,b shows the C1s spectra of GQDs and TiO_2_-GQDs caterpillar hybrid, which is fitted by four typical Gaussian peaks, and the centers are near 284.5 eV (P1), 286.1 eV (P2), 287.5 eV (P3), and 290.1 eV (P4). The peak located at the P1 corresponds to the C=C, which originated from the honeycomb lattice structure of sp^2^ hybridized carbon atoms. The peaks of P2, P3 and P4 are attributed to oxygen related functional groups in GQDs, respectively, which are C–O (ether) (P2), C=O (P3), and COOH (P4) [33,34,35]. It can be seen from the Figure 5b that the C–O and COOH peaks in the hybrid are reduced, which may be due to their poor stability. Therefore, unstable related functional groups can be transformed into in-plane epoxy groups to promote the formation of C–O–Ti bonds. The difference is that the edge carbon atoms bonded to C=O are highly stable and do not change during the formation of hybrid [36,37]. In addition, in Figure 5b, no functional groups related to Ti–C bond was found in the C1s spectrum of the TiO_2_-GQDs hybrid, which indicates that Ti and C are bonded by oxygen atom to form C–O–Ti covalent bonds [38]. The changes of surface states in TiO_2_-GQDs caterpillar hybrid samples are evaluated by the O1s XPS spectra, as Figure 5c,d show. The TiO_2_ caterpillar spectrum is fitted with two Gaussian peaks at binding energies of 529.7 and 531.2 eV, which are attributed to the lattice oxygen (Ti–O) [39,40] and surface hydroxyl group (Ti–OH) caused by the oxygen vacancies on the surface, respectively, suggesting the existence of adsorbed hydroxyl groups or water molecule [41]. In contrast, the O1s spectrum of TiO_2_-GQDs hybrids shows a difference. Three Gaussian peaks are fitted, which are located at 529.6 eV, 531.0 eV, and 535.5 eV, according to the XPS spectrum. Similarly, the peak centered at 529.6 eV is related to the oxygen in the crystal (Ti–O–Ti bond) [34]. In the literature [35,37,42], the XPS peaks in the range of 530.0–532.1 eV is usually attributed to the C–O–Ti bond; thus, it is considered that the strong peak at 531.0 eV comes from the C–O–Ti bond [38], which originates from the oxygen vacancy sites in TiO_2_ and in-plane oxygen functional (epoxy) groups in GQDs. Additionally, a new contribution with a maximum of 535.5 eV appeared, which is called hydroxyl functional group (C–OH) [36] when introducing GQDs. The Ti2p XPS spectra of the TiO_2_ caterpillar and TiO_2_-GQDs hybrid were also collected. The Ti2p consists of Ti^4+^ 2p_3/2_ (458.7 eV) and Ti^4+^ 2p_1/2_ (464.3 eV) spin-orbital as shown in Figure 5e, where it is typically referring to the characteristic of Ti^4+^-O bonds in TiO_2_ [43]. However, in the case of TiO_2_-GQDs caterpillar hybrids, along with Ti2p_3/2_ (458.4 eV) and Ti2p_1/2_ (464.1 eV) binding energies peaks, two new binding energy peaks at 457.7 eV and 463.4 eV are observed associated with Ti^3+^ valence states. The binding energy different (Δ) between (Ti^4+^ 2p_1/2_–Ti^4+^ 2p_3/2_) and (Ti^3+^ 2p_1/2_–Ti^3+^ 2p_3/2_) is 5.7 eV, which is similar to the report by previous study [44]. This unambiguously demonstrates the formation of Ti^3+^ valence states caused by oxygen vacancy during the process of ultrasound. Furthermore, the hybrids are slightly shifted towards lower binding energy compared with those in pure TiO_2_ caterpillar, suggesting that the chemical environment of Ti in the TiO_2_-GQDs has been changed due to the strong interaction of C–O–Ti bond between the TiO_2_ and GQDs. Therefore, the above results based on the XPS spectra indicate the existence of Ti^3+^, oxygen vacancies and C–O–Ti bond when coupled GQDs with TiO_2_ caterpillar, which may help to reduce the band gap of TiO_2_ and greatly promote the separation of photogenerated electrons and holes.

### 3.5. UV-Visible Absorption and Band Structure Studies

**Figure 6:**
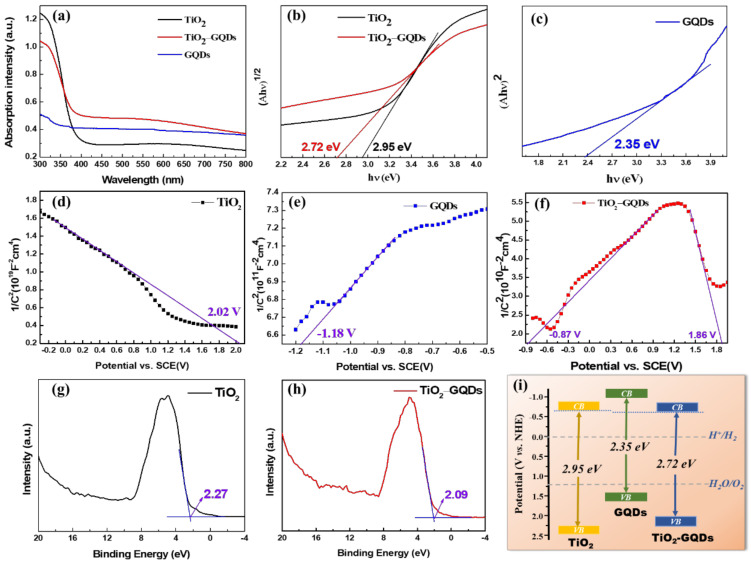
(**a**) UV-vis DRS. (**b**) The plots of (Ahν) ^1/2^ versus hν of TiO_2_ caterpillar and TiO_2_-GQDs hybrid samples and (**c**) the plots of (Ahν) ^2^ versus hν of GQDs. (**d**) M–S curves collected on TiO_2_ caterpillar, (**e**) GQDs and (**f**) TiO_2_-GQDs caterpillar hybrid. (**g**) VB XPS spectra of TiO_2_ caterpillar and (**h**) TiO_2_-GQDs hybrid. (**i**) Band structure diagram of GQDs, TiO_2_ caterpillar and TiO_2_-GQDs caterpillar hybrid.

As photocatalysts, the light absorption performance of samples is critical. The UV-vis absorption spectrum of GQDs, TiO_2_ caterpillar, and their hybrids samples are depicted in Figure 6a. In the spectrum of prepared GQDs, intense absorption in the UV region with the tail of the absorption band extending to the visible range and a weak absorption peak as a shoulder centered around 350 nm are associated with n→$\pi^{*}$ transition of C=O [27,45]. Therefore, this absorption spectrum is further evidence for the fabrication of GQDs. We can see that pristine TiO_2_ caterpillar nanoarchitecture can absorb most of the light in the UV region, and the absorption edge wavelength is around 420 nm, while the absorption edge of TiO_2_-GQDs hybrid sample is visibly red-shifted due to the synergistic effect between TiO_2_ and GQDs, showing stronger absorption in the visible region. The expansion of light absorption range is beneficial to visible light photocatalysis. This indicates that the incorporation of GQDs reduces the band gap relative to pure TiO_2_ caterpillar. The band gap narrowing and extended absorption should be attributed to the hybrid formation through the chemical bonding between TiO_2_ and GQDs, i.e., the formation of C–O–Ti covalent bonds, similar to carbon-doped TiO_2_ composites. Under light irradiation, this C–O–Ti bond will facilitate the efficient interfacial charge transfer from GQDs to the TiO_2_ caterpillar, thus prolonging the absorption of the hybrid in the visible region, resulting in the improved photocatalytic performance of the TiO_2_-GQDs. Furthermore, the optical bandgap energy (*E_g_*) of the samples could be estimated by the transformational Tauc’s plot obtained from the Kubellka–Munk function [46]:

(1) $$\left(\alpha h\nu \right)=A{\left(h\nu -E_{g}\right)}^{n/2},$$

where *α* is the absorption coefficient, *h* is Planck’s constant, *A* is a constant, *ν* is the light frequency, and *n* = 1 and 4 for direct and indirect band gap materials, respectively. The optical transitions of TiO_2_ are indirect and the value of *n* is thus 4 [47], while *n* = 1 for GQDs [18]. Figure 6b shows the plot of (*αhν*)^1/2^ versus *hν* of TiO_2_ caterpillar and TiO_2_-GQDs hybrid. Using the Tauc plot, we estimated the *E_g_* of TiO_2_ and TiO_2_-GQDs caterpillar nanostructure to be around 2.95 and 2.72 eV, respectively. This is further proof that when TiO_2_ caterpillar is combined with GQDs, its band gap reduces, and thus the response range in visible light is improved. Similarly, Figure 6c shows the *E_g_* of GQDs is around 2.35 eV.

To gain insights into the intrinsic properties and effects of GQDs on the band structure of the TiO_2_, the flat band potentials (*V_fb_*) of GQDs, TiO_2_ caterpillar and TiO_2_-GQDs caterpillar hybrid were measured by M–S plots, which can be estimated from the plots based on Equation (2) [48]:

(2) $$\frac{1}{C^{2}}=\left(\frac{2}{\varepsilon_{r}\varepsilon_{0}eN_{d}A^{2}}\right)\left[\left(V-V_{fb}\right)-\frac{kT}{e}\right],$$

where *C* is the specific capacity and *V_fb_* is the flat band potential. It can be seen that the reciprocal of the square of capacity (1/*C*^2^) has a linear relationship with the applied potential (*V*), and take them as *Y*-axis and *X*-axis, respectively, as shown in Figure 6d–f, the intercept of the line on the *X*-axis is the *V_fb_*. Furthermore, the *V_fb_* and the positive or negative slope of the line can be used to estimate the position of the conduction band (CB) or valence band (VB), respectively. For example, the negative slope of the tangent line in Figure 6d indicates the highest potential of the VB can be extremely close to its *V_fb_*. As a result, the *V_fb_* of TiO_2_ caterpillar was calculated to be 2.02 V vs. SCE (2.26 V vs. standard hydrogen electrode (NHE)). In Figure 6e, the tangent line with a positive slope indicates the lowest potential of CB could be estimated from its *V_fb_* accordingly. Therefore, the CB bottoms of the GQDs were confirmed to be −1.18 V vs. SCE (−0.94 V vs. NHE). Furthermore, combined with *E_g_* of GQDs in Figure 6c, the VB values of GQDs were calculated to be 1.41 V vs. NHE. An inverted V-shape is shown in Figure 6f, indicating that a heterojunction forms at the interface between TiO_2_ caterpillar and GQDs with distinct electronic behavior. Thus, the CB and VB in the TiO_2_-GQDs caterpillar hybrids were determined to be −0.87 V vs. SCE (−0.63 V vs. NHE) and 1.86 V vs. SCE (2.10 V vs. NHE), respectively [49,50]. In order to better elucidate the energy band structure of photocatalysts, the exact VB positions for TiO_2_ caterpillar and TiO_2_-GQDs caterpillar hybrid were also verified by VB XPS measurement, which can give information related to the total density of states (DOS) of the VB [48,51]. As shown in Figure 6g,h, the maximum VB energies of TiO_2_ and TiO_2_-GQDs caterpillar hybrid were estimated to be at 2.27 eV and 2.09 eV, respectively, indicating the alteration of the electronic structure after introducing GQDs. Meanwhile, combined with the *E_g_* in Figure 6b, the minimum CB energies may be obtained to be at about −0.68 eV for TiO_2_ caterpillar and −0.63 eV for TiO_2_-GQDs caterpillar hybrid. Both of the results conform well to the *V_fb_* values estimated in Figure 6d,f. Therefore, according to the analysis of the energy band positions of the above different samples, the band structures of TiO_2_ caterpillar and TiO_2_-GQDs caterpillar hybrid compared to the potentials for water reduction and oxidation were well resolved, as illustrated in Figure 6i [52,53]. It was concluded that GQDs, TiO_2_, and TiO_2_-GQDs all have appropriate energy level positions, which meet the thermodynamic condition for photocatalytic splitting of water toward hydrogen evolution. Compared to pristine TiO_2_, a more positive CB of TiO_2_-GQDs caterpillar hybrid may reduce thermionic emission induced by high potential barrier height of the photocatalyst-Pt contact [49].

### 3.6. Photocatalytic Properties Studies

**Figure 7:**
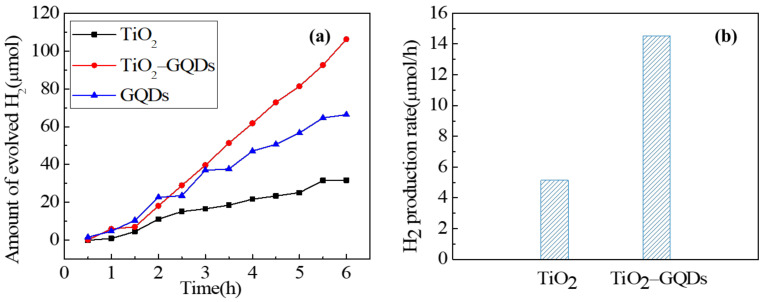
(**a**) Amount of photocatalytic H_2_ production, and (**b**) average photocatalytic H_2_ production rate for different samples under simulated solar light irradiation.

In order to study the photocatalytic performance of TiO_2_ caterpillar after introducing GQDs, the photocatalytic water reduction for hydrogen production under simulated solar light irradiation using TiO_2_ caterpillar, GQDs, and TiO_2_-GQDs hybrid were further investigated. Since Pt is a traditional and efficient co-catalyst, H_2_PtCl_6_ is used as a precursor to reduce the overpotential required for water decomposition to hydrogen production [54]. From Figure 7a, it can be seen that hydrogen production of all samples increases with the extension of irradiation time. After 360 min of light irradiation, total hydrogen evolution over pure TiO_2_ caterpillar and GQDs reached 30 and 70 μmol, respectively. In contrast, the photocatalytic H_2_ evolution activity of TiO_2_-GQDs caterpillar hybrid was significantly enhanced, which reached the maximum H_2_ production of 110 μmol. Additionally, as Figure 7b shows, the pure TiO_2_ caterpillar exhibited a relatively low hydrogen production rate (5.2 μmol/h); this may be because the absorption range of TiO_2_ is limited to UV light and a small part of visible light, and the photogenerated charge carriers recombine quickly. After coupling with GQDs, the H_2_ generation rate of hybrid was the highest (18.3 μmol/h), which is about 3.5 times the H_2_ production rate of pure TiO_2_ caterpillar. Moreover, the H_2_ evolution rate of pure caterpillar and hybrid are higher than that of P25 (2.3 μmol/h) and CQDs/P25 (9.1 μmol/h) reported by Yu et al. [55] and are also higher than that of pure TiO_2_ (0.3 μmol/h) and TiO_2_/GQDs (2.2 μmol/h) photocatalysts published by Min et al. [6]. The above results show that the combination of pure TiO_2_ and GQDs is an effective means to improve the activity of photocatalytic H_2_ production. Due to the strong coupling between TiO_2_ caterpillar and GQDs, the electron-hole pairs at the interface between GQDs and TiO_2_ are effectively separated through the C–O–Ti bonds, resulting in high photocatalytic H_2_ evolution activity. In addition, there is hot electron injection from GQDs to TiO_2_ caterpillar, which is also conducive to photocatalytic performance [56].

### 3.7. Analysis of Photocatalytic Hydrogen Generation Mechanism

**Figure 8:**
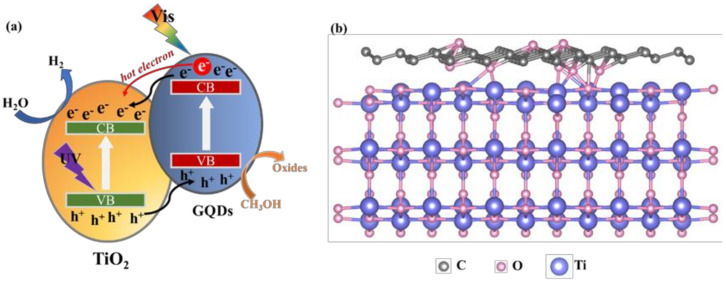
(**a**) Schematic of interfacial carrier transfer mechanism of photocatalytic hydrogen production for TiO_2_-GQDs caterpillar hybrid. (**b**) Schematic model for the C–O–Ti bond.

Based on the above results, a possible interfacial charge transfer mechanism of photocatalytic hydrogen production for TiO_2_-GQDs caterpillar hybrid is illustrated in Figure 8a. The TiO_2_-GQDs heterojunction forms a type-II (staggered gap) band alignment, which is more thermodynamically favorable. First, since the incorporation of GQDs reduces the band gap and the synergistic effect between TiO_2_ caterpillar and GQDs, both UV and visible region of incident light can be utilized simultaneously in the TiO_2_-GQDs hybrid to produce electron-hole pairs. TiO_2_ can absorb the UV part of the incident light, while GQDs absorb visible light. Second, the attractive heterostructure can lead to charge separation at the interface, where GQDs and TiO_2_ act as donor and acceptor, respectively. The possible C–O–Ti bond was formed by rearranging epoxy functional groups in GQDs and oxygen vacancies in TiO_2_ caterpillar, which further enhanced the interfacial electron transfer from GQDs to TiO_2_. A schematic model for the C–O–Ti bond in hybrid is presented in Figure 8b. Another key factor is the existence of hot electron injection from GQDs to TiO_2_ caterpillar due to the strong interaction between them. Finally, once the charges are separated, the CB electrons of TiO_2_ which transferred from GQDs and generated by TiO_2_ will migrate to the surface and react with water molecules to produce H_2_. In addition, the highly ordered 3D TiO_2_ caterpillar nanoarchitecture can shorten the lateral diffusion pathways of charge carriers to the surface due to its large specific surface area and promote better dispersion of GQDs to extend the number of active sites. As a result, the introduction of GQDs not only extends the visible light response region but also increases the charge separation rate in order to improve the photocatalytic hydrogen production capacity of TiO_2_ caterpillar. The entire photocatalytic reaction process can be described by the following equations [45]:

(3) $$photocatalyst+hv\to e^{-}+h^{+},$$

(4) $$h^{+}+H_{2}O\to \cdot OH+H^{+},$$

(5) $$CH_{3}OH+\cdot OH\to \cdot CH_{2}OH+H_{2}O,$$

(6) $$\cdot CH_{2}OH\to HCHO+H^{+}+e^{-},$$

(7) $$2H_{2}O+2e^{-}\to H_{2}+2OH^{-},$$

(8) $$\mathrm{overall}\;\mathrm{reaction}:\;CH_{3}OH\to HCHO+H_{2},\;$$

## 4. Conclusions

In summary, the designed TiO_2_-GQDs caterpillar hybrid was employed to study its photocatalytic water reduction for hydrogen production. It is proposed that ultrasound can induce the formation of oxygen vacancies in TiO_2_ caterpillars, and the rearrangement of in-plane epoxy functional groups in GQDs, such that they can form hybrids through the possible C–O–Ti bonds. The addition of GQDs to TiO_2_ caterpillar results in a stronger visible light absorption capability and the well-matched band-structure of the TiO_2_-GQDs hybrid theoretically satisfies the water photo-splitting for hydrogen production. The hydrogen evolution rate of TiO_2_-GQDs hybrid can reach 18.3 μmol/h, which is over 3.5 times that of pure TiO_2_. The enhanced photocatalytic activity of hybrid may be attributed to the unique morphology of caterpillar nanoarchitecture and rapid interfacial electron transfer from GQDs to TiO_2_ via the C–O–Ti bonds. This work will contribute to the development of low-cost and high-efficiency photocatalysts with unique morphology and provide ideas for more fields of research.

## Data Availability

The data presented in this study are available on request from the corresponding author.
